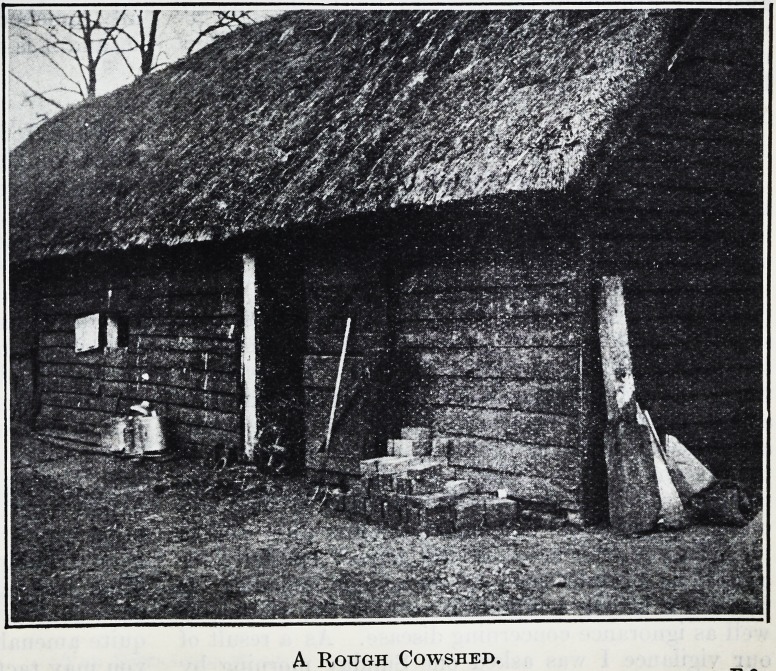# Clean Milk without Expense

**Published:** 1924-09

**Authors:** 


### CLEAN MILK WITHOUT EXPENSE.

In a pamphlet issued by the Ministry of Agriculture which has been written by the staff of the National Institute for Research in Dairying, University College, Reading, under the title "Studies concerning the Handling of Milk" (Miscellaneous Publications, No. 41, Stationery Office, 1s. net.), stress is laid upon the immense importance of the personal factor in ensuring a clean milk supply. It is also demonstrated that great improvements in the supply of milk can be secured without embarking on any serious expense.

THE COWSHED REMODELLED.

The interesting fact emerges that farm workers, not properly instructed in the cleansing of covered pails, were actually getting worse results with them than with open pails :—

Buckets, open and covered, were washed by the farm hands with warm water and a cloth in accordance with the usual practice. The milk taken into the covered bucket contained many more bacteria than that taken into the open bucket. When, however, the clean milker who was engaged for the experiment washed the bucket, this difference disappeared, although the clean milker was also using warm water and the same cloth for the work. The clean milker understood what she was aiming at; the farm workers did not, and as it was a little more difficult to see the angles in the covered bucket, they were insufficiently cleansed and rapidly accumulated a layer of dried milk.

Experiments in what could be done with a small outlay were made by the staff of the Institute when it came into possession of a farm in 1920 :—

The shed was unevenly cobbled and badly lit. There was no dairy, but an abundant supply of water could be obtained from a hand pump in the yard. The workers washed and stored their utensils in the position shown in the photograph. The utensils were steamed in the cowshed itself just before milking took place, and hot water taken from the steamer was used to assist the washing up after the milking was finished. The work was done under considerable disadvantages. Nevertheless the condition of the milk was converted from very average to clean. At a small cost standings for the cows were put in, and a gutter made. The thatched roof still remains, but is kept clean and lime spray is freely used. . . . The photograph demonstrates how greatly these small improvements have affected the lighting and look of the interior of the shed. A calf shed across the yard has been converted into a dairy by putting in an impervious floor and a few wooden fittings The water is still carried by hand across the yard, galvanised baths are used for washing up, and a simple steam sterilizer is used for the sterilization of all utensils.

The whole of the alterations are of a very modest description, but make the work much pleasanter, and a great improvement in the milk supply is the result. The photographs referred to are here reproduced by the courtesy of the Ministry of Agriculture. It is uphill work getting into the head of the farmer the importance of steam sterilization of all utensils. There is a very large avoidable loss in the souring of milk every year, yet sterilization involves little expense.

A ROUGH COWSHED.

## Figures and Tables

**Figure f1:**
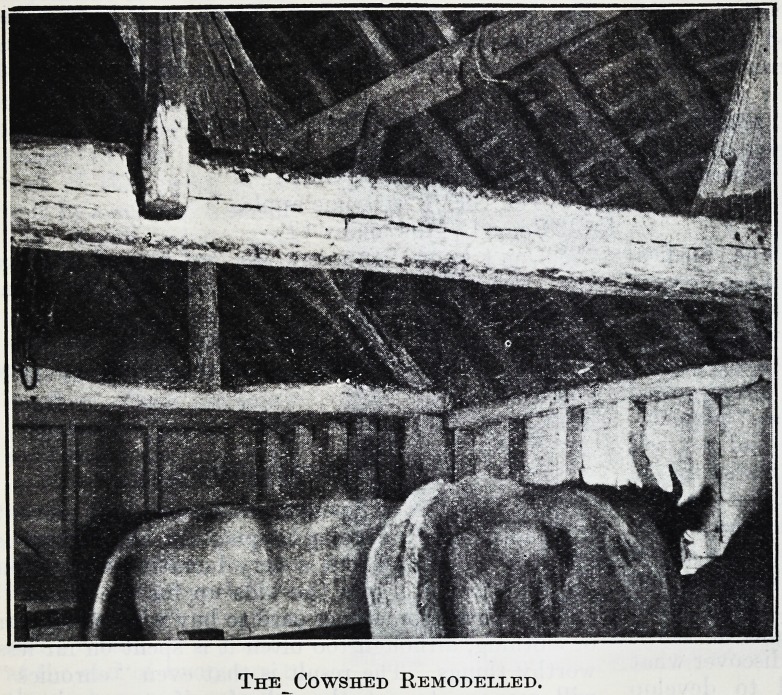


**Figure f2:**